# Critical Insights for Neutrophil-to-Lymphocyte Ratio and Platelet-to-Lymphocyte Ratio in Paediatric Systemic Lupus Erythematosus

**DOI:** 10.31138/mjr.070325.dhr

**Published:** 2025-06-30

**Authors:** Seher Şener

**Affiliations:** Department of Paediatric Rheumatology, Sophia Children’s Hospital, Erasmus University Medical Centre, Rotterdam, The Netherlands

**Keywords:** neutrophil-to-lymphocyte ratio, paediatric, platelet-to-lymphocyte ratio, systemic lupus erythematosus

Dear Editor,

We read with great interest Nath et al.’s study on the potential role of the neutrophil-to-lymphocyte ratio (NLR) and platelet-to-lymphocyte ratio (PLR) in evaluating disease activity in paediatric systemic lupus erythematosus (pSLE).^1^ The authors suggest that NLR and PLR may serve as trusted and cost-effective tools for assessing and predicting disease activity in pSLE. While the study presents compelling findings, we believe that certain gaps and limitations merit further consideration.

One important aspect not fully explored is the potential influence of treatment regimens on NLR and PLR. Treatment strategies in pSLE often include corticosteroids, conventional disease-modifying anti-rheumatic drugs (DMARDs), and biologic therapies, all of which may impact these ratios.^2^ Investigating how these therapies affect NLR and PLR could provide valuable insights into their potential as predictive biomarkers, considering the variability in treatment response.

Additionally, the study relies solely on the Systemic Lupus Erythematosus Disease Activity Index 2000 (SLEDAI-2K) to assess disease activity.^3^ While this widely accepted tool is valuable, incorporating additional measures such as the Physician’s Global Assessment or the Systemic Lupus International Collaborating Clinics/American College of Rheumatology (SLICC/ACR) damage index could offer a more comprehensive evaluation of disease progression.^4,5^ Furthermore, assessing the correlation between NLR and PLR with specific organ involvement would be advantageous, given the diverse manifestations of pSLE across different organ systems.

Lastly, the absence of longitudinal follow-up in this study raises questions about the prognostic utility of NLR and PLR. A longitudinal study, incorporating repeated measures of NLR and PLR, could enhance our understanding of how these ratios fluctuate with disease course, periods of remission, and flare-ups, offering potential as long-term predictors in patients with pSLE.

In conclusion, while the study makes an important contribution to the field, we suggest that future research address these gaps by including larger, more diverse populations, evaluating treatment impacts, incorporating additional disease activity measures, and introducing longitudinal follow-up. This would enhance the clinical utility of NLR and PLR as biomarkers in pSLE.

## AUTHOR CONTRIBUTIONS

SS conceived the study, designed the research, conducted the literature review, collected, and analysed the data, and wrote the manuscript.

## CONFLICT OF INTEREST

The author has indicated no potential conflicts of interest to disclose.

## FUNDING

No specific funding was received from public, commercial or not-for-profit bodies to carry out the work described in this article.

## ETHICS APPROVAL AND WRITTEN INFORMED CONSENT STATEMENTS

Not applicable.
